# Effect of expansion media and fibronectin coating on growth and chondrogenic differentiation of human bone marrow-derived mesenchymal stromal cells

**DOI:** 10.1038/s41598-021-92270-4

**Published:** 2021-06-22

**Authors:** Valentina Basoli, Elena Della Bella, Eva Johanna Kubosch, Mauro Alini, Martin J. Stoddart

**Affiliations:** 1 Regenerative Orthopaedics, AO Research Institute Davos, Clavadelerstrasse 8, Davos Platz, Switzerland; 2 Department of Orthopedics and Trauma Surgery, Faculty of Medicine, Medical Center-Albert-Ludwigs-University of Freiburg, Albert-Ludwigs-University of Freiburg, 79106 Freiburg, Germany

**Keywords:** Mesenchymal stem cells, Multipotent stem cells, Stem-cell differentiation

## Abstract

In the field of regenerative medicine, considerable advances have been made from the technological and biological point of view. However, there are still large gaps to be filled regarding translation and application of mesenchymal stromal cell (MSC)-based therapies into clinical practice. Indeed, variables such as cell type, unpredictable donor variation, and expansion/differentiation methods lead to inconsistencies. Most protocols use bovine serum (FBS) derivatives during MSC expansion. However, the xenogeneic risks associated with FBS limits the use of MSC-based products in clinical practice. Herein we compare a chemically defined, xenogeneic-free commercial growth medium with a conventional medium containing 10% FBS and 5 ng/ml FGF2. Furthermore, the effect of a fibronectin-coated growth surface was investigated. The effect of the different culture conditions on chondrogenic commitment was assessed by analyzing matrix deposition and gene expression of common chondrogenic markers. Chondrogenic differentiation potential was similar between the FBS-containing αMEM and the chemically defined medium with fibronectin coating. On the contrary, the use of fibronectin coating with FBS-containing medium appeared to reduce the differentiation potential of MSCs. Moreover, cells that were poorly responsive to in vitro chondrogenic stimuli were shown to improve their differentiation potential after expansion in a TGF-β1 containing medium. In conclusion, the use of a xenogeneic-free medium provides a suitable alternative for human bone marrow MSC expansion, due the capability to maintain cell characteristic and potency. To further improve chondrogenic potential of BMSCs, priming the cells with TGF-β1 during expansion is a promising strategy.

## Introduction

The regenerative medicine field requires a cell source for either tissue engineering or secretome production. Ideally, any novel therapy should be efficient, fast, and cost-effective, maintaining the original physiological functions in the process^1,2^. Given this ambitious expectation for the development of in vitro tissues, there is also an increasing research interest for fabrication of customized, patient specific implants, while other branches in this field are focusing on the use of cell products, such as secretome or extracellular vesicles, as a future strategy for tissue healing^3^. Many of these processes require an initial step of primary cell expansion. Regenerative medicine typically uses patient's autologous cells, seeds them on suitable support materials, induces cell differentiation through the addition of specific growth factors and then uses the matured construct or the cell secretome to induce healing or for a total replacement of the tissue^4,5^.

Although research in this field is rapidly progressing, there are still numerous and fundamental questions to be answered: which is the optimal cell type^6^, how many cells are needed^7^, and what is the most suitable method of cell isolation, proliferation and differentiation that can guarantee a high yield of commitment into a stable phenotype. These factors are highly affected by donor variability^8–10^, as well as the culture methods^11,12^.

So far, research in this field has tested countless sources of mesenchymal stromal cells (MSCs) from different sites such as bone marrow, dental pulp^13^, adipose tissue^14^, and synovial membrane^15^, demonstrating that it is possible to isolate and differentiate them towards different phenotypes. However, most of the techniques described use fetal bovine serum (FBS) to support cell growth during expansion. While on one hand it supports cell growth and differentiation, on the other hand FBS is an animal-derived product, complicating the translation of any biotech therapy^16^. Studies have highlighted the possibility that proteins and peptides contained in FBS can be incorporated into cells during the transfection process, consequently causing a rejection of transplanted cells^17,18^. Moreover, FBS is an ill-defined culture media component, being a heterogeneous solution of growth factors and proteins that are not controllable and are subject to batch-to-batch variation. This variability between different lots of the same product is recognized to influence cell proliferation and phenotype^19^.

In this study, we therefore aim to compare the growth and differentiation of primary human bone marrow MSCs (BMSCs) expanded in standard FBS-containing αMEM medium or in a commercial serum-free medium. The FBS containing medium was further supplemented with Fibroblast Growth Factor -2 (FGF2) as this factor is known to maintain cells in a more stem-like state and promotes proliferation^20–22^. The combination of MSC-screened FBS with FGF2 is generally considered to be the "gold standard" expansion medium for primary human MSCs. We further investigated the potential of Transforming Growth Factor (TGF)-β priming in monolayer cultures to enhance BMSC chondrogenic potential. In addition, according with previous studies performed on MSCs derived from adipose tissue, bone marrow, and umbilical cord^23^, we tested whether using fibronectin to coat the culture surface, in combination with the different media, can improve chondrogenic differentiation of primary human BMSCs.

## Results

### Effect of different expansion protocols on BMSC growth and morphology

**Figure 1:**
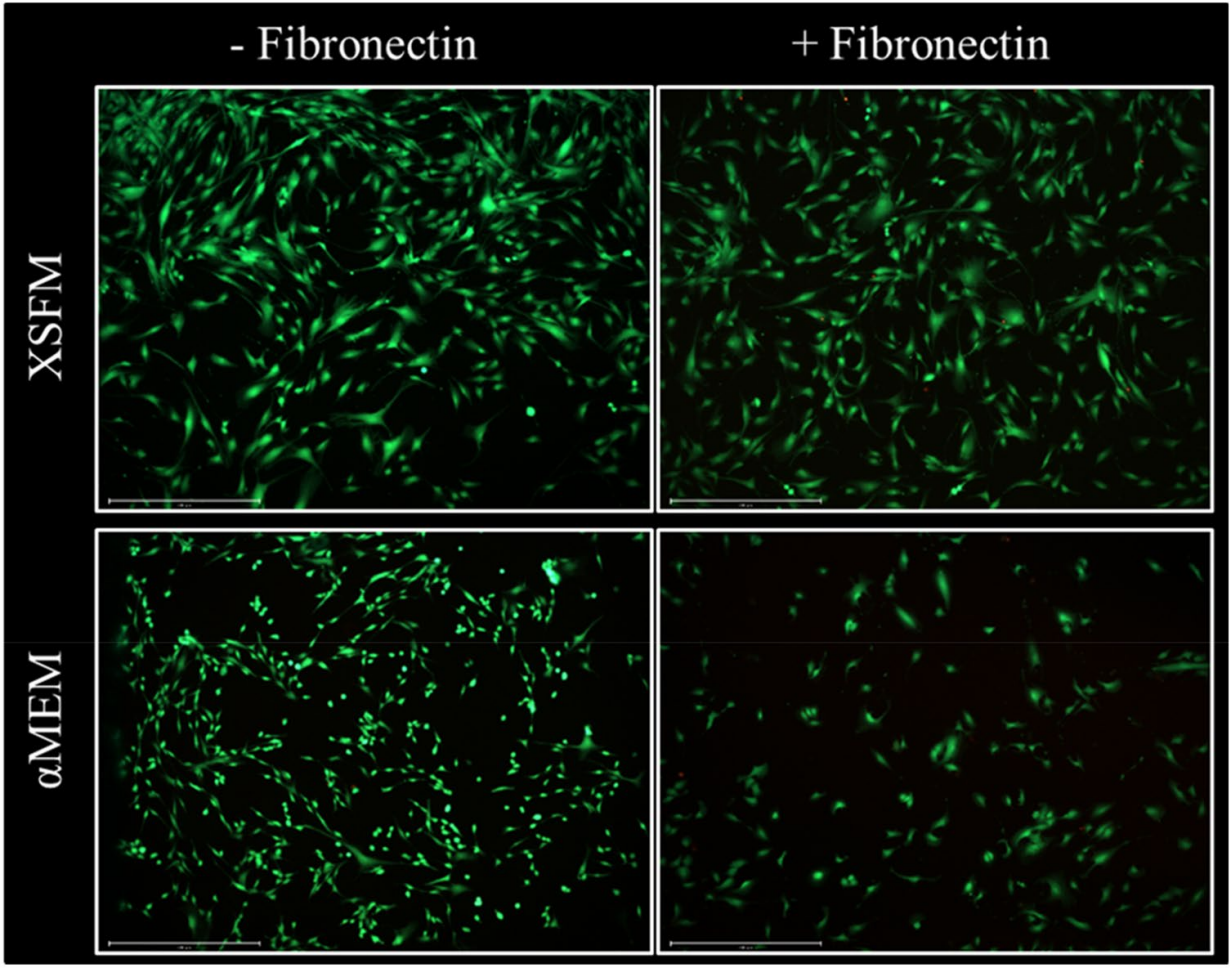
Live/Dead assay of BMSCs seeded on a glass chamber slide, with or without fibronectin coating, and cultured with αMEM/FBS/FGF2 or with a chemically defined medium (XSFM) for 72 h. Green: Calcein-AM that stains live cells; red: ethidium homodimer for dead cells. Scale bar (bottom left in each panel): 200 µm. Figure representative of n = 5 biological replicates.

As expected, media composition did not affect cell viability, as assessed by Live/Dead staining. The presence or absence of a fibronectin coating also had no apparent effect, even though the combination of fibronectin coating with αMEM/FBS/FGF2 appears to result in a lower number of cells (Fig. 1).

**Figure 2:**
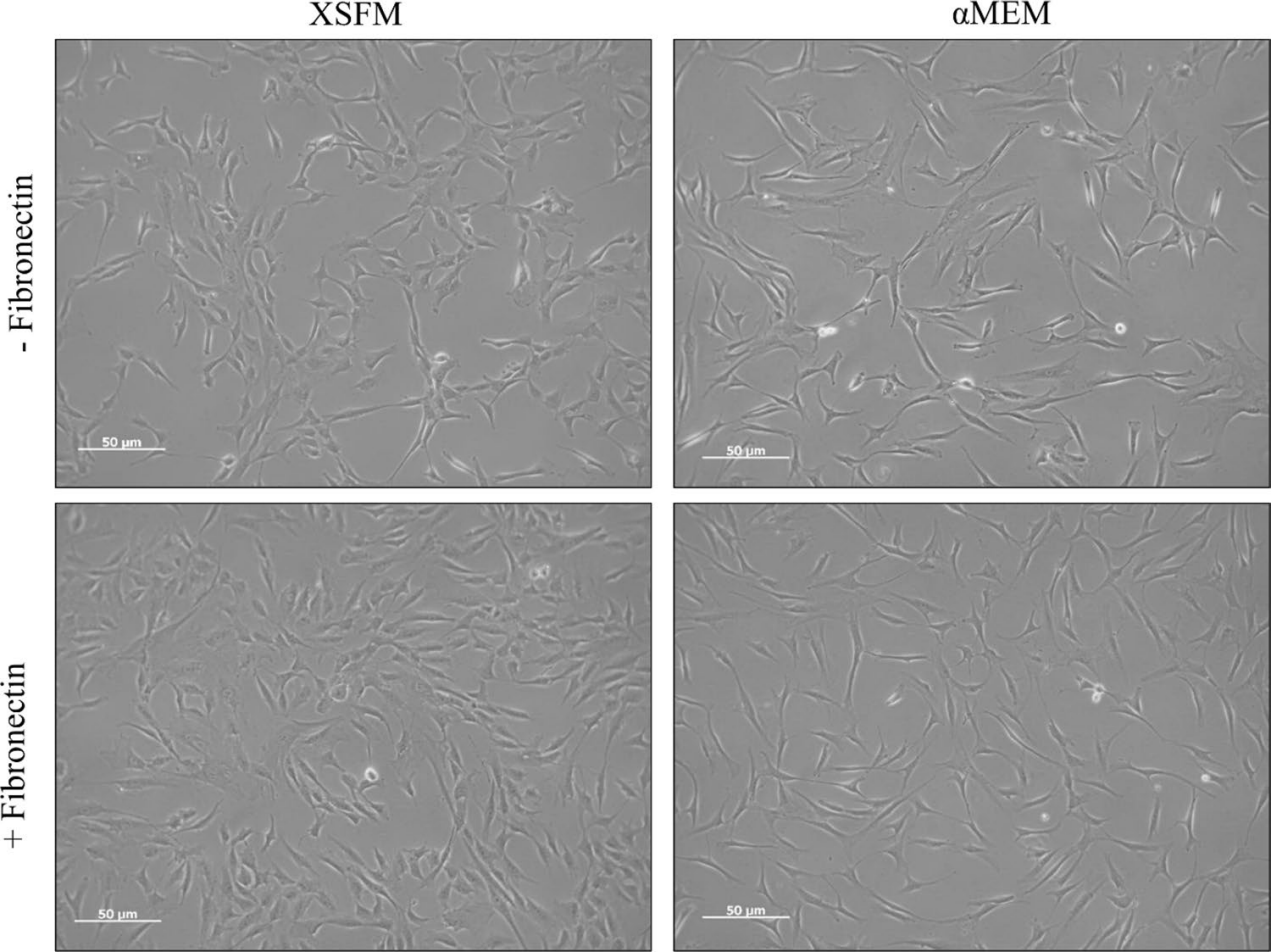
Representative images of BMSCs seeded on tissue culture plastic, with or without a 5 μg/ml fibronectin coating and cultured with αMEM/FBS/FGF2 or XSFM for 72 h. The Figure is representative of n = 5 biological replicates.

All cells in the presence of a chemically defined medium (xeno/serum free medium—XSFM) or αMEM exhibited a fibroblast-like and spindle-shaped morphology with long cell processes (Fig. 2).

**Figure 3:**
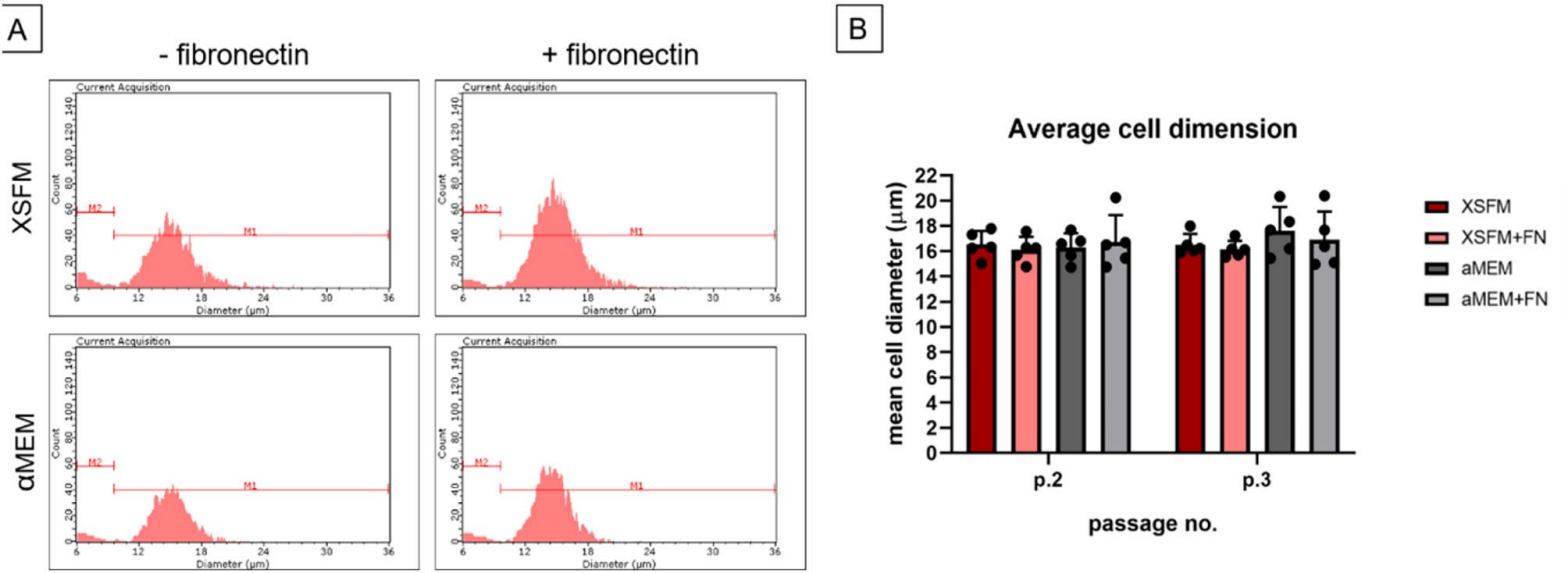
Analysis of cell dimension of BMSCs grown in αMEM/FBS/FGF2 or XSFM, with or without fibronectin coating. (**A**) Examples of histograms that can be obtained with using Scepter 2.0 for cell counting representative for 1 donor. X-axis reports cell diameter in µm, while the y-axis reports the count of cells. M1 gate includes intact cells larger than 9.6 μm, while M2 includes cell debris (6–9.6 μm). The average cell dimension for each cell population in (**B**) is calculated from M1. The comparison between growth conditions revealed no significant difference in cell average cell diameter (n = 5 donors).

Cell size was measured in two consecutive passages: the results showed no significant variation of the average cell diameter for BMSCs expanded in the different conditions, with a higher variability observed in αMEM/FBS/FGF2 groups (Fig. 3).

**Figure 4:**
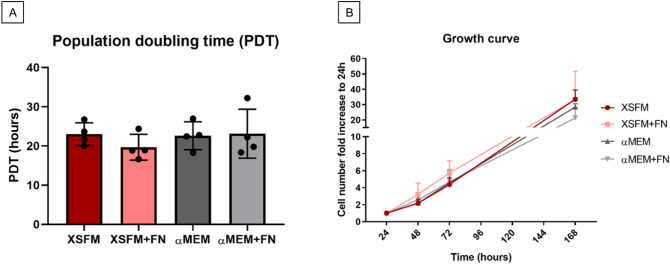
Cell growth and metabolic activity of BMSC cultured in αMEM/FBS/FGF2 (grey shades) or XSFM (red shades) up to 168 h (7 days). Tissue culture plastic was either pre-coated with 5 μg/ml fibronectin (light color lines and bars) or not (dark color lines and bars). (**A**) Comparison of BMSC doubling time (range 24–72 h) among the different groups, expressed in hours. (**B**) Growth curve indicating the average cell fold increase referred to 24 h after cell adhesion. n = 4 donors.

The population doubling time (PDT) was similar for cells grown in XSFM or in αMEM/FBS/FGF2, with no statistically significant differences among the groups (Fig. 4A). The PDT was calculated in the range 24–72 h for the cells to be in the exponential phase of cell growth. The growth curve also showed a similar trend, with no difference between the groups between 24 and 72 h (Fig. 4B). As expected, the cells reached a growth plateau at day 7, with similar cell numbers for all groups.

### Chondrogenic differentiation of BMSCs expanded in αMEM/FBS/FGF2 or XSFM

**Figure 5:**
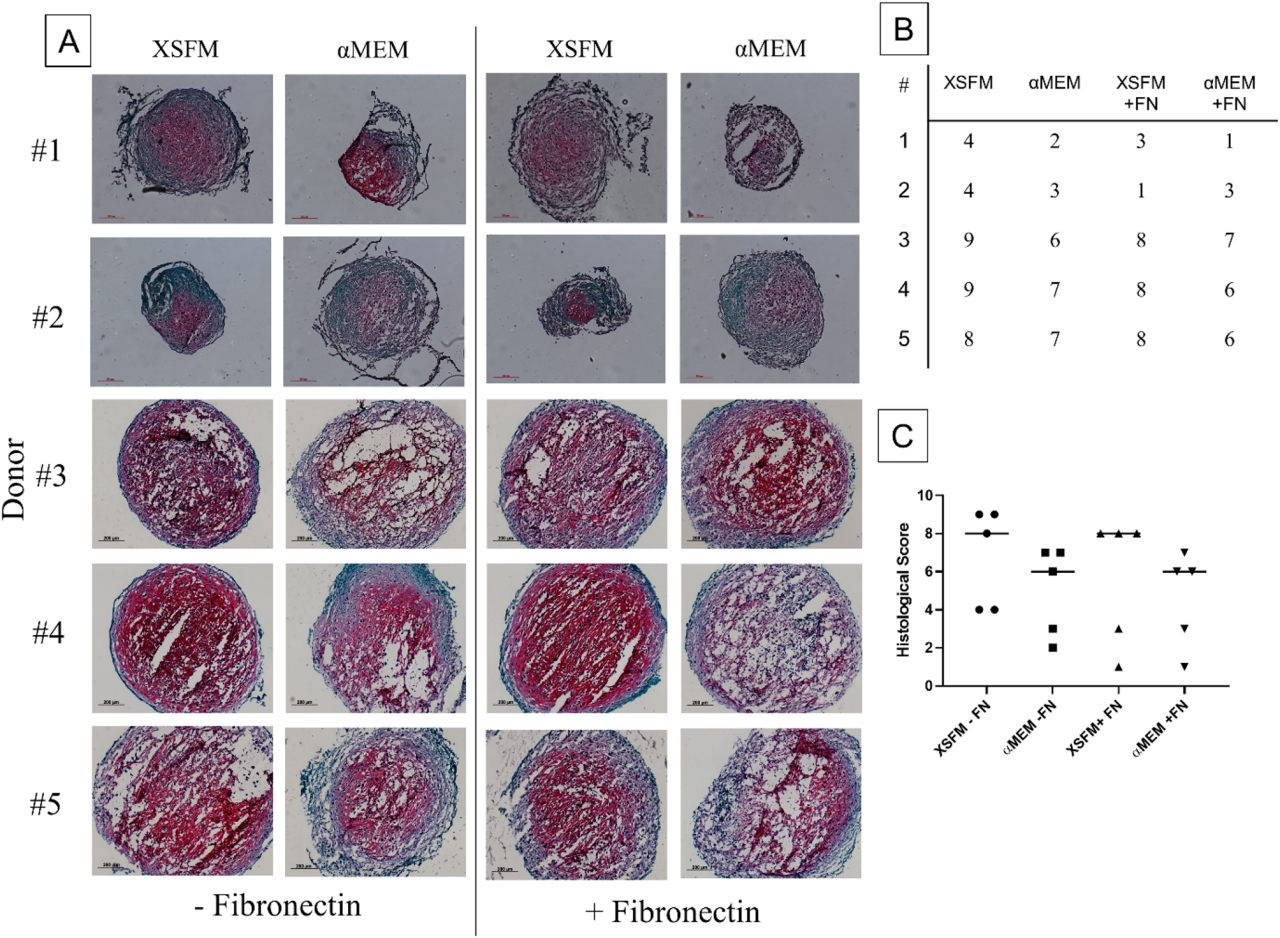
Analysis of the chondrogenic potential of BMSCs expanded in different cell culture media (n = 5 donors). (**A**) Representative images of pellet cryosections stained by Safranin-O/Fast Green: the intensity of Safranin-O (red) staining is directly proportional to the extracellular proteoglycan content, while Fast Green solution (green) stains collagen fibers. Scale bar (bottom left in each picture) = 200 µm. (**B**) The table indicates the histological score assigned to each pellet (1- undifferentiated; 10- very good differentiation). Grading of the safranin O staining was ranked on a scale of 1–10 according to the Bern Score^24^. (**C**) Comparison of the histological scores among the groups for all donors.

After comparing cell growth in different media, we focused on the chondrogenic differentiation potential of BMSCs after expansion in different conditions. We used two different types of donors that can be classified as good responders (n = 3) or poor responders (n = 2), based on histological results of cells expanded with the standard medium (αMEM/FBS/FGF2, no fibronectin coating, Fig. 5A–C).

Chondrogenic differentiation was evaluated at 21 days by Safranin-O/Fast Green staining of pellet cryosections (Fig. 5A). The histological evaluation for all donors expanded with XSFM did not show any difference compared to αMEM/FBS/FGF2 medium, although there was a tendency towards a stronger intensity for the matrix deposition and internal pellet structure either with or without fibronectin. Pellets from all groups had comparable diameters (data not shown). However, pellets from BMSCs expanded with αMEM/FBS/FGF2 on a fibronectin coating showed in some cases (donors #1, #4 and #5) a considerable dispersion and poor matrix organization compared to the other groups, indicating a greater variability.

The use of XSFM with BMSC from non-responsive donors improved the size of differentiated pellets, but not the intensity for Safranin-O staining.

**Figure 6:**
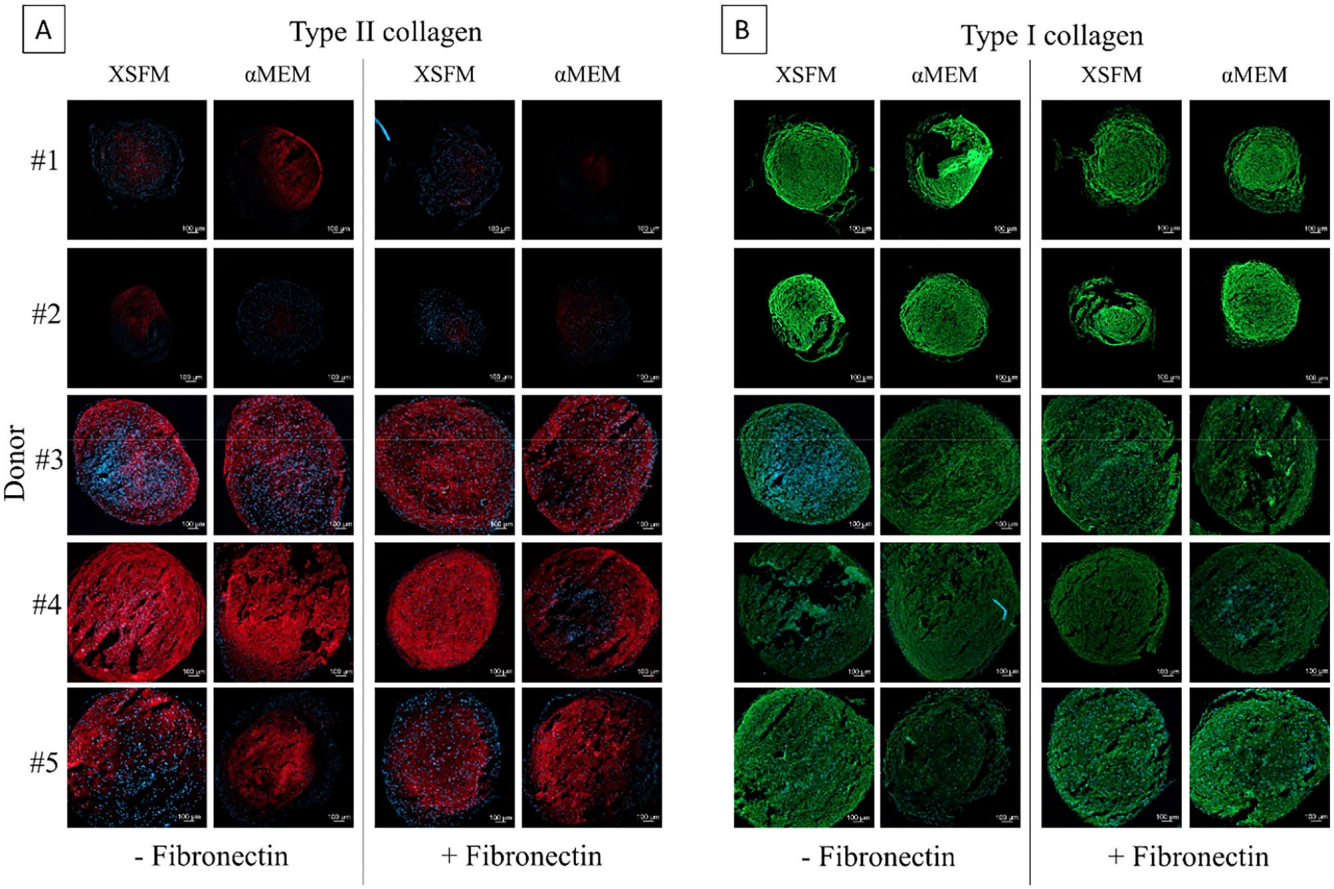
Immunohistological analysis of the chondrogenic potential of BMSCs expanded in different cell culture media (n = 5 donors). Pellet cryosections stained for type II (**A**) and type I collagen (**B**). The intensity of fluorescent secondary antibody is directly proportional to the production of different collagen content, while DAPI (blue) stains cell nuclei. Scale bar (bottom right in each picture) = 100 µm.

To determine the quality of matrix produced in hBMSC pellets, immunofluorescence staining for type II and I collagen was performed. The use of XSFM or αMEM medium did not influence the deposition of type II collagen within the donors. Donors #1 and #2, as previously observed with Saf-O/Fast-Green staining (Fig. 5), were less responsive to the differentiation induced by TGF-β1, and immunofluorescence confirmed that there was almost no production of type II collagen. The other 3 donors did not show differences in type II collagen staining after cell expansion with different media (Fig. 6A). Type I collagen staining also did not show any particular influence of the expansion medium or fibronectin coating on matrix deposition. Interestingly, the less-responsive donors showed a higher staining intensity for type I collagen compared to the other donors (Fig. 6B).

### Gene expression analysis on chondrogenic markers

**Figure 7:**
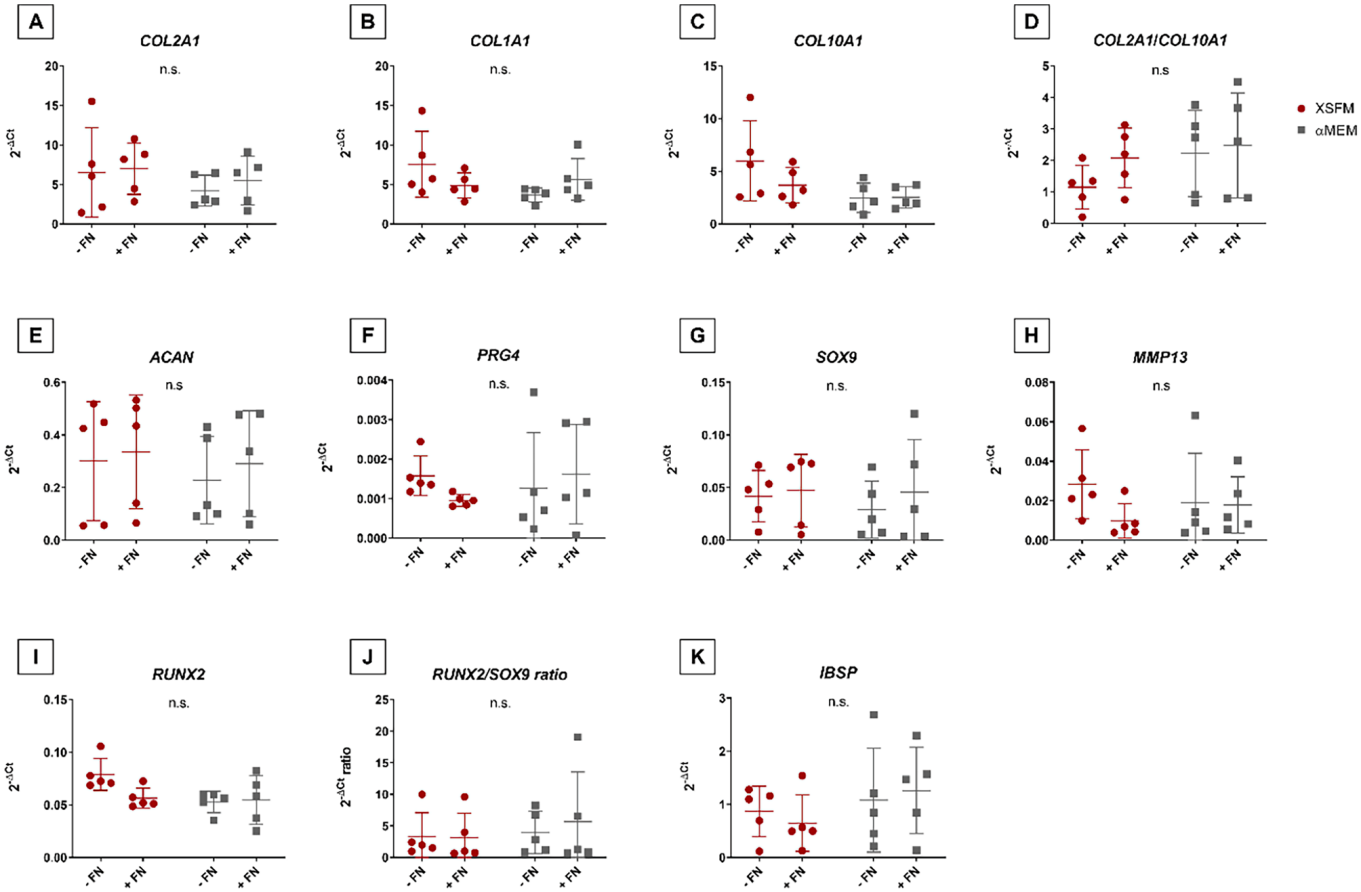
Culture conditions during monolayer expansion of BMSCs modulate differently the expression of chondrogenic markers during differentiation. After expansion in either αMEM/FBS/FGF2 or XSFM (with or without fibronectin coating), cells were cultured for 21 days in chondrogenic medium containing 10 ng/ml of TGF-β1. The expression of (**A**) *COL2A1*, (**B**) *COL1A1*, (**C**) *COL10A1*, (**D**) *COL2A1*/*COL10A1* ratio, (**E**) *ACAN*, (**F**) *PRG4*, (**G**) *SOX9*, (**H**) *MMP13,* (**I**) *RUNX2,* (**J**) *RUNX2/SOX9* ratio and (**K**) *IBSP* were normalized to ribosomal protein large, P0 (*RPLP0*). The gene expression levels are represented as 2^(-ΔCt)^ (mean ± SD; n = 5). Grey bars represent scatterplot of different donor measurements in BMSC cultured in αMEM, red bars represent scatterplot of different measurements in BMSC cultured in XSFM medium, FN is the presence ( +) or absence (−) fibronectin coating during the BMSC expansion. Data are expressed as mean ± SD.

Histological evaluation showed that the use of XSFM medium during in vitro expansion led to similar differentiation when compared to medium containing serum and 5 ng/ml FGF2. We further investigated the expression of common molecular markers associated with chondrogenic differentiation. Analysis of cartilage related genes expression (*COL2A1, COL1A1, COL10A1*, *ACAN, PRG4 and SOX9*, Fig. 7A–G) showed no difference between groups, although a positive trend in XSFM expanded cells compared to αMEM/FBS/FGF2, except for PRG4 (Fig. 7F), can be observed. Similarly, αMEM/FBS/FGF2 tends towards an increase in *COL2A1/COL10A1* ratio, but this did not reach significance (Fig. 7D). The expression of cartilage hypertrophy-associated markers, namely *COL10A1* and *MMP13* (Fig. 7C,H), were also similar between different groups, with no statistically significant differences. We further studied the genes associated to osteogenic differentiation, *RUNX2, IBSP* (Fig. 7I,K) and the balance of chondrogenesis and osteogenesis though* RUNX2/SOX9* ratio (Fig. 7J). Results showed no significant differences among groups of media and the use or not of fibronectin for* RUNX2* expression and *RUNX2/SOX9* ratio. The expression of* IBSP* (Fig. 7K) showed an increased trend for the group αMEM/FBS/FGF2 compared to XSFM, but it was not statistically significant.

Overall, the results from histological evaluations, as well as from gene expression analysis, showed that cells in a chemically defined medium displayed similar subsequent chondrogenic differentiation.

### Non-responsive donor priming

**Figure 8:**
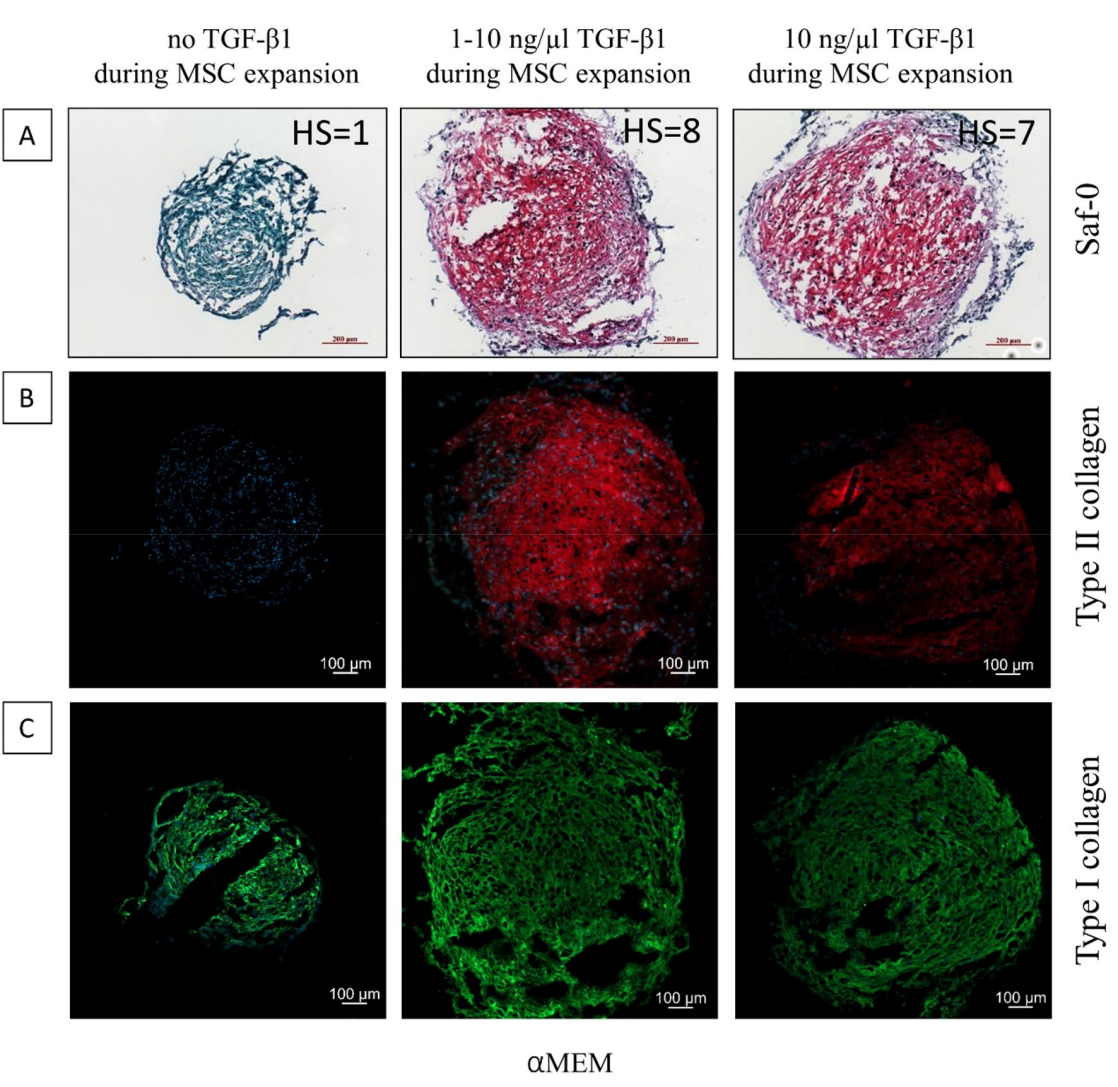
Representative images of Safranin-O/Fast Green staining on chondrogenic pellets of poorly responsive cells (n = 2 donors); here it is shown donor #1 (**A**). BMSCs were cultured in αMEM/FBS/FGF2 with different concentrations of TGF-β1 during cell expansion. The intensity of Safranin-O (red) staining is directly proportional to the proteoglycan content inside the pellet, while Fast Green counterstains collagen fibers. Cells in monolayer were expanded in the presence of increasing concentration of TGF-β1 (1–10 ng/ml) or with constant 10 ng/ml of TGF-β1 before the induction of chondrogenesis. All the cells were induced to chondrogenic differentiation using the same medium containing 10 ng/ml TGF-β1. Each image includes the histological score (HS) assigned on the top right corner. Scale bar for 10 × Objective = 200 µm (bottom-right in each panel). (**B, C**) Immunohistological analysis of the chondrogenic potential of BMSCs expanded in αMEM supplemented or not with 1–10 ng/ml or 10 ng/ml TGFβ during MSC expansion. Pellet cryosections stained for type II (**B**) and type I (**C**) collagen. The intensity of staining is directly proportional to the collagen content, while DAPI (blue) stains cell nuclei. Scale bar (bottom left in each picture) = 100 µm.

In a separate experiment, we tested the effect of adding TGF-β1 to the standard expansion medium on subsequent chondrogenesis, in the attempt to improve differentiation of poorly responsive cells. Specifically, poorly responsive cells were expanded in αMEM/FBS/FGF2 as previously described. However, in the final 5 days of expansion, TGF-β1 was added either at increasing concentration (1 to 10 ng/ml) or at 10 ng/ml. Cell priming with gradual increase or constant concentration of TGF-β1 during the expansion in monolayer in αMEM strongly improved chondrogenic differentiation (Fig. 8A) in terms of qualitative scoring, compared to cells expanded without the addition of TGF-β1 in monolayer cultures (n = 2). These findings show that the use of TGF-β1 during cell expansion can be beneficial for chondrogenesis and can have a pivotal role in priming non-responsive donors.

As previously observed by Saf-O/Fast-Green staining, BMSCs cultured with or without TGF-β1 differently responded to chondrogenic differentiation in pellet culture. Indeed, the use of 1–10 ng/ml and 10 ng/ml TGF-β1 significantly improved the yield of type collagen II production in non-responsive donors, specifically in the picture donor #1 is shown (Fig. 8B). Previously we observed (Fig. 6A,B) that the donors with a poor type II collagen deposition had a high type I collagen content; here the non-responsive donors primed using TGF-β1 did not show a massive change in the type I collagen staining intensity, with a slight reduction in fluorescence intensity in the 10 ng/ml TGF-β1 group (Fig. 8C).

## Discussion

Human-derived mesenchymal stem cells (MSCs) are a promising cell source for clinical applications in regenerative medicine, due their relative ease of harvest^25^. However, one of the most difficult tasks in cell manufacture is controlling their quality.

FBS has been used as a primary component of cell expansion media for decades^26,27^, principally because it promotes adhesion and cellular expansion. However, due to several problems associated with batch-to-batch variability, uncontrolled composition that may vary from supplier to supplier, as well as the risk of zoonosis and transmissions of proteins not desired at the cellular level, its use is still challenging in a Good Manufacturing process (GMP) environment. One of the reasons why the application of MSCs in regenerative medicine appears to be limited is due to the non-standardized expansion protocols used in various labs. Methods involving the use of platelet lysates have been proposed^28^, but also this alternative is affected by innumerable variables, mostly associated to the donor variation^29^. In our study we have shown that the use of a chemically defined medium without FBS (XSFM) has the same performance as the gold standard medium containing FBS and 5 ng/ml FGF2 starting with the same condition of cells isolated and then cryopreserved in the presence of FBS. This suggests XSFM medium as a clinically relevant alternative.

Fibronectin coating has been proposed as a beneficial method to enhance MSC adhesion onto plastic surfaces^30^. Fibronectin does not limit cell growth in XSFM medium; on the contrary, cells expanded in αMEM/FBS/FGF2 were negatively affected in terms of growth.

We investigated the expression of main chondrogenic markers after 21 days of differentiation, concluding that the use of XSFM medium did not modify the cell potency, indeed no significant difference in the expression of the main chondrogenic markers was observed compared with αMEM/FBS/ 5 ng/ml FGF2.

Duan et al. previously demonstrated that TGF-β and BMP rapidly mobilizes additional TGFBRs from intracellular stores to the cell surface, increasing the abundance of cell-surface TGFBRs in HaCaT and A549 cell lines, priming the downstream TGF-β signaling^31^. Based on this report and on our previous work on the TGFBR expression profile^32^, we investigated the potential of late addition of TGF-β1 into the expansion medium as a means to improve chondrogenic differentiation. Poorly responsive cells, after expansion in our standard medium, were grown again in αMEM/FBS/FGF2 and in the final week of expansion either increasing concentrations of TGF-β1 or a constant 10 ng/ml TGF-β1 was added. We observed that there was a significant enhancement of chondrogenic differentiation in terms of matrix deposition. This enhancement was similar to that seen in previous studies using siRNA against *TGFBR2*^32^, thus offering a powerful method to modulate MSC phenotype that is easily applied. This also demonstrates that cell phenotype can be modified even in late stages of expansion. Therefore, TGF-β1 addition during the expansion process can prime chondrogenic differentiation, probably by enhancing the downstream pathways associated with Small Mother Against Decapentaplegic (SMAD) proteins. Indeed, other studies have highlighted the role of TGF-β signaling associated with recruitment of the ALK5 (*TGFBR1*) receptor, with the consequent activation SMAD 2/3 pathway, while excess of TGF-β can lead to activation of ALK1 (*ACVRL1*), promoting the activation of SMAD 1/5/8^33^. We know from previous studies that these receptors are present on MSCs and are essential for chondrogenesis^34^.

Furthermore, it has been shown that increasing TGF-β concentration shifts the balance of ALK signaling from ALK5 (SMAD 2/3) at low dose to ALK1 (SMAD 1/5/8) at higher doses^35^. Further analysis is ongoing to understand the possible mechanism during the priming of MSC and the role of TGF-β1 in the expansion medium.

The main limitation of this study resides in the use of previously banked BMSCs. As described in the materials and methods section, our procedure of BMSC isolation is highly standardized to minimize manipulation-dependent sources of variation. The procedure involves the use of a tested batch of MSC-qualified FBS and the cryopreservation at Passage 1 in an FBS/DMSO solution. For this reason, this study was conducted with cells that were exposed to xenoderivatives during the isolation phase and first duplications in vitro. Future studies will be focused on analyzing more in detail the effect of different media composition during the isolation phase, where the presence of a fibronectin coating might be essential, and the overall performance of cells which were never treated with any animal-derived products.

In summary, this report describes how the use of xenogeneic serum-free medium during BMSC expansion provides the same performance compared to FBS containing medium with respect to chondrogenic commitment. Furthermore, the use of TGF-β1 during the later stages of cell expansion strongly improves the yield of differentiation in those donors defined as less responsive to TGF-β1 stimulation. With the vision of clinical translation, the use of serum-free medium could pave the way to future application in the clinical scenario by allowing chemically defined cell culture, thus limiting variations among different FBS batches. In addition, a xenogeneic serum-free medium avoids a potential risk for developing zoonosis in patients treated with cultured cells. The use of serum-free medium that has the same performance as the gold standard represents a step further for the use of cells, tissue engineered constructs and cell related products that can be suitable for the treatment of pathological and traumatic situations.

## Methods

All methods were performed in accordance with the relevant guidelines and regulations.

### Isolation and expansion of human mesenchymal stem cells from bone marrow

Bone marrow from 5 different donors was harvested from vertebral body after signed informed consent and full ethical approval (Ethic -Commission, Albert-Ludwigs-University Freiburg, Germany, AN-EK-135/14). Fresh bone marrow was diluted 1:4 and layered on top of Histopaque-1077 solution (Sigma-Aldrich), in a proportion of 2.6 ml of Histopaque per ml of undiluted marrow. After centrifugation at 800 g for 20 min, the mononuclear cell-containing interface was recovered, and cells were counted using the Cell Scepter 2.0 Automated Cell Counter (Millipore). Isolated cells were seeded at a density of 50,000 cells/cm^2^ into 300 cm^2^ tissue culture flasks in Minimum Essential Medium Eagle, Alpha Modification (αMEM; Gibco, Thermo Fisher, Zürich, Switzerland) containing 10% batch-tested FBS for MSC expansion (PAN-Biotech, Aidenbach, Germany), 100 U/mL penicillin, and 100 μg/mL streptomycin (Gibco), and 5 ng/ml recombinant human basic Fibroblast Growth Factor (FGF2, Fitzgerald Industries International, Acton, MA, USA). Cells were maintained at 37 °C in 5% CO_2_, 95% humidity atmosphere. After 4 days, non-adherent hematopoietic cells were removed to select the BMSC population. Medium was then refreshed every 2nd day. Cells at passage 1 were frozen in 8% dimethyl sulfoxide (DMSO) and 92% FBS.

After thawing, and during the cell expansion phase, two different media were compared. One was αMEM medium containing 10% FBS and 5 ng/ml FGF2 described above for cell isolation (αMEM/FBS/FGF2), while the second was a chemically defined medium (PRIME-XV MSC Expansion XSFM, Fujifilm Irvine Scientific—hence XSFM). Where specified, tissue culture plastic was coated with a fibronectin solution (5 µg/ml fibronectin in PBS—Fujifilm Irvine Scientific, Santa Ana, CA, USA) according to the manufacturer's protocol.

In summary, BMSCs were expanded under the following conditions:

- αMEM/FBS/ 5 ng/ml FGF2
- αMEM/FBS/ 5 ng/ml FGF2 with fibronectin coating
- XSFM
- XSFM with fibronectin coating.

Cells were cultured until passage 3 in the different conditions. At each passage, the cells were counted, and their size measured using the Cell Scepter 2.0 Automated Cell Counter (Millipore), which uses the Coulter principle for particle analysis. The average cell size (expressed as diameter) was recorded and analyzed as a quantitative morphological cell parameter.

In a separate experiment, αMEM/FBS/FGF2 medium was supplemented with the addition of increasing TGF-β1 concentrations (Fitzgerald, using 1 ng/ml for 48 h, 5 ng/ml for the following 48 h, then 10 ng/ml for the final 24 h) or constant 10 ng/ml TGF-β1 for the final 5 days before chondrogenic commitment, in order to improve chondrogenesis of BMSCs.

A schematic of the experimental design is represented in Supplementary Fig. 1.

### Live/dead assay

Viability of cells seeded at 3000/cm^2^ on glass chamber slides (Falcon) was determined by Live/Dead staining. After 72 h, cells were stained with 2 mM Calcein AM (Invitrogen, Thermo Fisher) and 4 mM ethidium homodimer-1 (EthD-1, Invitrogen, Thermo Fisher) in culture medium for 30 min and immediately evaluated by confocal microscopy (LSM800, Zeiss AG, Feldbach, Switzerland).

### Cell counting and population doubling

Proliferation of BMSCs expanded according to different protocols was measured by counting cells with a Trypan Blue exclusion test after 24 h, 48 h, 72 h and 7 days after seeding at p.2. Briefly, cells were seeded at a density of 3000/cm^2^ in 6-well tissue culture plates and cultured with αMEM or XSFM medium, with or without a fibronectin coating. After cell detachment with 0.05% Trypsin-Ethylenediaminetetraacetic acid (EDTA) (Gibco), the cells were counted in a Neubauer hemocytometer after a two-fold dilution in Trypan Blue.

The population doubling time (PDT) was calculated using the following formula:

$$PDT = ~\left( {t2 - t1} \right)~ \times ~\frac{{\log 2}}{{\log \frac{{N2}}{{N1}}}}$$

where t is time (hours), N is the number of cells, and N2 and N1 represent the number of cells at t2 (72 h) and t1 (24 h), respectively. The PDT was calculated in the range 24–72 h for the cells to be in the exponential phase of cell growth.

At 72 h, cell cultures were imaged for a qualitative assessment of cell morphology.

### Chondrogenic differentiation

Chondrogenic differentiation of BMSCs was performed in 3D pellet culture. 2 × 10^5^ BMSCs per pellet were seeded in V-bottom 96-well plates (Corning, Corning, NY, USA). Cells were centrifuged for 5 min at 400 g in either chondropermissive (for undifferentiated control) or chondrogenic medium. Chondropermissive medium contained Dulbecco's Modified Eagle's Medium (DMEM) 4.5 g/l glucose (Gibco), 100 U/mL penicillin, and 100 μg/mL streptomycin (Gibco), 1% non-essential amino acids (Gibco), 1% ITS + (Corning); chondrogenic medium was obtained with the addition of 100 nM dexamethasone (Sigma-Aldrich, Buchs, Switzerland), 10 ng/ml TGF-β1 (Fitzgerald Industries International), and 50 µg/ml ascorbic acid-2 phosphate (Sigma-Aldrich). The medium was replaced every second day and pellets were harvested for further analyses after 21 days.

### Real-time quantitative PCR analysis

Total RNA was isolated from adherent hBMSC cells during passaging and from 3D chondrogenic pellets at day 0 (cells before cell seeding) and after 21 days of differentiation using TRI Reagent Solution (Molecular Research Center Inc., Cincinnati, OH, USA) according to the manufacturer’s protocol. RNA quantity was measured using a NanoDrop 1000 Spectrophotometer (Thermo Fisher). For reverse transcription, TaqMan Reverse Transcription reagents (Applied Biosystems, Thermo Fisher) were used. The reverse transcription reaction was initiated at 25 °C for 10 min, followed by 30 min at 42 °C and ended by 5 min at 85 °C. qPCR reactions were set up in 10 μl reaction mixtures containing TaqMan Gene Expression Master Mix (Applied Biosystems, Thermo Fisher), gene-specific assays (Supplementary Table 1), Diethyl pyrocarbonate (DEPC)-H_2_O and 10 ng of cDNA template. qPCR analysis was performed in a QuantStudio 7 Flex Real-Time PCR System (Thermo Fisher). The reaction program was set up as follows: 50 °C for 2 min, 95 °C for 10 min and 40 cycles of 95 °C for 15 s followed by an annealing/extension step at 60 °C for 1 min. Triplicates were used for each target gene (technical replicates). The relative expression of *SOX9*, *ACAN*, *MMP13*, *COL2A1*, *COL10A1, COL1A1, PRG4, SOX9, RUNX2 and IBSP* during chondrogenic differentiation was determined using the 2^(-ΔCt)^ method, with ribosomal protein large, P0 (*RPLP0*) as reference gene and day 0 RNA as the calibrator sample.

### Histological staining

#### Safranin-O/ fast green

At day 21, pellets were fixed in 70% methanol, then transferred to sucrose-PBS 5% solution overnight, before being embedded in cryocompound (Thermo Fisher). Cryosections were cut with a thickness of 8–10 µm. For Safranin-O staining, samples were first stained with Weigert’s Haematoxylin for 10 min, followed by 6 min with Fast Green and 15 min with Safranin-O solutions. After dehydration with increasing concentrations of ethanol and xylene, samples were coverslipped with Eukitt mounting medium and dried overnight.

The histological sections were observed using Zeiss AxioPlan 2 Microscope (Zeiss Microscopy GmbH, Jena, Germany) with objective 10X/0.50. Pictures were acquired using RGB camera 1X (16 bit) and Axiocam Software (Zeiss Microscopy GmbH, Göttingen, Germany). Grading of the safranin O staining was on a scale of 1–10 as assessed according with the Bern Score^24^.

#### Immunofluorescence

Cryosections were washed for 10 min with dH_2_O to remove the cryocompound, transferred to absolute methanol for 20 min and washed twice in 0.01% Tween 20 in PBS (PBS-T). Enzymatic treatment by 1U/ml Hyaluronidase (Sigma-Aldrich, H3506) and 0.25 U/ml Chondroitinase ABC (Sigma-Aldrich, C2905) in PBS-T for 30 min at 37 °C allowed the digestion of matrix. After washing in PBS-T, pellets were transferred in blocking solution containing 5% horse serum (Vector laboratories, S-2000) in PBS-T for 30 min at room temperature. Primary antibody anti-type II collagen (4 μg/mL, CIICI, see acknowledgement section) and anti-type I collagen (1:400, Origene Acris, R1038) were added over night at 4 °C. Slides were washed with PBS-T, then the secondary antibodies were added, 4 μg/ml anti Rabbit Alexa Fluor 488 against type I collagen (Thermo Fisher, A-11008) and 5 μg/ml anti Mouse Alexa Fluor 660 against type II collagen (Thermo Fisher, A-21055) for 1 h at 37 °C. After washing with PBS-T, the nuclei were counterstained with 2-(4-Amidinophenyl)-1H-indole-6-carboxamidine (DAPI) 2.5 μg/mL and then cover slipped with ProLong mounting solution (Thermo Fisher, P10144).

### Statistical analysis

A total number of 5 donors were used for chondrogenesis experiments, while 4 were used for cell counting/population doubling calculations. Statistical analysis was performed using GraphPad Prism 7.03 software. Non-parametric two-way Analysis of Variance (ANOVA) with Tukey's multiple comparison test were applied, with *p* < 0.05 considered as statistically significant. A two-way ANOVA was used to evaluate the distributions and homogeneity variance in the groups.

## Supplementary Information

Supplementary Information 1.

Supplementary Information 2.

## Data Availability

All data and materials used in the analysis are available to any researcher for purposes of reproducing or extending the analysis.

## Abbreviations

αMEM: Minimum essential medium Eagle, alpha modification
ANOVA: Analysis of variance
FGF2: Basic fibroblast growth factor
BMSCs: Bone-marrow MSCs
DEPC: Diethyl pyrocarbonate
DMEM: Dulbecco's modified Eagle's medium
DMSO: Dimethyl sulfoxide
EDTA: Ethylenediaminetetraacetic acid
EthD-1: Ethidium homodimer-1
FBS: Foetal bovine serum
MSCs: Mesenchymal stromal cells
PDT: Population doubling time
SMAD: Small mother against decapentaplegic
TGF-β: Transforming growth factor, beta
XSFM: Xeno-serum-free medium

## References

[1] Mason C, Dunnill P (2008). A brief definition of regenerative medicine. Regen. Med..

[2] Griffith LG, Naughton G (2002). Tissue engineering–current challenges and expanding opportunities. Science.

[3] Szychlinska MA (2019). Functional biomolecule delivery systems and bioengineering in cartilage regeneration. Curr. Pharm. Biotechnol..

[4] Monaco G, El Haj AJ, Alini M, Stoddart MJ (2021). Ex vivo systems to study chondrogenic differentiation and cartilage integration. J. Funct. Morphol. Kinesiol..

[5] Szychlinska MA (2020). Evaluation of a cell-free collagen type i-based scaffold for articular cartilage regeneration in an orthotopic rat model. Materials.

[6] Jorgensen C, Gordeladze J, Noel D (2004). Tissue engineering through autologous mesenchymal stem cells. Curr. Opin. Biotechnol..

[7] Langer R (2009). Perspectives and challenges in tissue engineering and regenerative medicine. Adv. Mater..

[8] Alhadlaq A, Mao JJ (2004). Mesenchymal stem cells: isolation and therapeutics. Stem. Cells Dev..

[9] Siddappa R, Licht R, van Blitterswijk C, de Boer J (2007). Donor variation and loss of multipotency during in vitro expansion of human mesenchymal stem cells for bone tissue engineering. J. Orthop. Res..

[10] Stocum DL (2001). Stem cells in regenerative biology and medicine. Wound Repair Regen..

[11] Mirbagheri M (2019). Advanced cell culture platforms: a growing quest for emulating natural tissues. Mater. Horiz..

[12] Liu S (2017). Manufacturing differences affect human bone marrow stromal cell characteristics and function: comparison of production methods and products from multiple centers. Sci. Rep..

[13] Maioli, M. *et al.* Osteogenesis from dental pulp derived stem cells: a novel conditioned medium including melatonin within a mixture of hyaluronic, butyric, and retinoic acids. *Stem Cells Int.* **2016** (2016). 10.1155/2016/2056416 PMC4736970 26880937

[14] Basoli V (2017). Melatonin and vitamin d interfere with the adipogenic fate of adipose-derived stem cells. Int. J. Mol. Sci..

[15] Kovermann NJ (2019). BMP2 and TGF-beta cooperate differently during synovial-derived stem-cell chondrogenesis in a dexamethasone-dependent manner. Cells.

[16] Rathore AS, Winkle H (2009). Quality by design for biopharmaceuticals. Nat. Biotechnol..

[17] Gregory CA, Reyes E, Whitney MJ, Spees JL (2006). Enhanced engraftment of mesenchymal stem cells in a cutaneous wound model by culture in allogenic species-specific serum and administration in fibrin constructs. Stem Cells.

[18] Spees JL (2004). Internalized antigens must be removed to prepare hypoimmunogenic mesenchymal stem cells for cell and gene therapy. Mol. Ther..

[19] Lipsitz YY, Timmins NE, Zandstra PW (2016). Quality cell therapy manufacturing by design. Nat. Biotechnol..

[20] Hoch AI, Leach JK (2014). Concise review: optimizing expansion of bone marrow mesenchymal stem/stromal cells for clinical applications. Stem Cells Transl. Med..

[21] Mossahebi-Mohammadi M, Quan M, Zhang J-S, Li X (2020). FGF signaling pathway: a key regulator of stem cell pluripotency. Front. Cell Dev. Biol..

[22] Bianchi G (2003). Ex vivo enrichment of mesenchymal cell progenitors by fibroblast growth factor 2. Exp. Cell Res..

[23] Vanda S, Ngo A, Tzu Ni H (2018). A xeno-free, serum-free expansion medium for ex-vivo expansion and maintenance of major human tissue-derived mesenchymal stromal cells. Transl. Biomed..

[24] Grogan SP (2006). Visual histological grading system for the evaluation of in vitro-generated neocartilage. Tissue Eng..

[25] Szychlinska MA (2020). Cycloastragenol as an exogenous enhancer of chondrogenic differentiation of human adipose-derived mesenchymal stem cells: a morphological study. Cells.

[26] Mizukami A, Swiech K (2018). Mesenchymal stromal cells: from discovery to manufacturing and commercialization. Stem Cells Int..

[27] Leffert H (1974). Growth control of differentiated fetal rat hepatocytes in primary monolayer culture: V: occurrence in dialyzed fetal bovine serum of macromolecules having both positive and negative growth regulatory functions. J. Cell Biol..

[28] Muraglia A (2017). Culture medium supplements derived from human platelet and plasma: cell commitment and proliferation support. Front Bioeng. Biotechnol..

[29] Cho HS (2011). Individual variation in growth factor concentrations in platelet-rich plasma and its influence on human mesenchymal stem cells. Korean J. Lab. Med..

[30] Ogura N (2004). Differentiation of the human mesenchymal stem cells derived from bone marrow and enhancement of cell attachment by fibronectin. J. Oral Sci..

[31] Duan D, Derynck R (2019). Transforming growth factor–β (TGF-β)–induced up-regulation of TGF-β receptors at the cell surface amplifies the TGF-β response. J. Biol. Chem..

[32] Rothweiler R (2020). Predicting and promoting human bone marrow MSC chondrogenesis by Way of TGFbeta receptor profiles: toward personalized medicine. Front Bioeng. Biotechnol..

[33] Davidson ENB (2009). Increase in ALK1/ALK5 ratio as a cause for elevated MMP-13 expression in osteoarthritis in humans and mice. J. Immunol..

[34] Hellingman CA (2011). Smad signaling determines chondrogenic differentiation of bone-marrow-derived mesenchymal stem cells: inhibition of Smad1/5/8P prevents terminal differentiation and calcification. Tissue Eng. Part A.

[35] Remst DF (2014). TGF-ss induces Lysyl hydroxylase 2b in human synovial osteoarthritic fibroblasts through ALK5 signaling. Cell Tissue Res..

