# Supplementary figures and images for: Effect of expansion media and fibronectin coating on growth and chondrogenic differentiation of human bone marrow-derived mesenchymal stromal cells
Source: Sci Rep. 2021 Jun 22;11:13089. doi: 10.1038/s41598-021-92270-4 (PMC8219706; doi:10.1038/s41598-021-92270-4)

**Supplementary Figure 1**. Schematic representation of the experimental design.

**
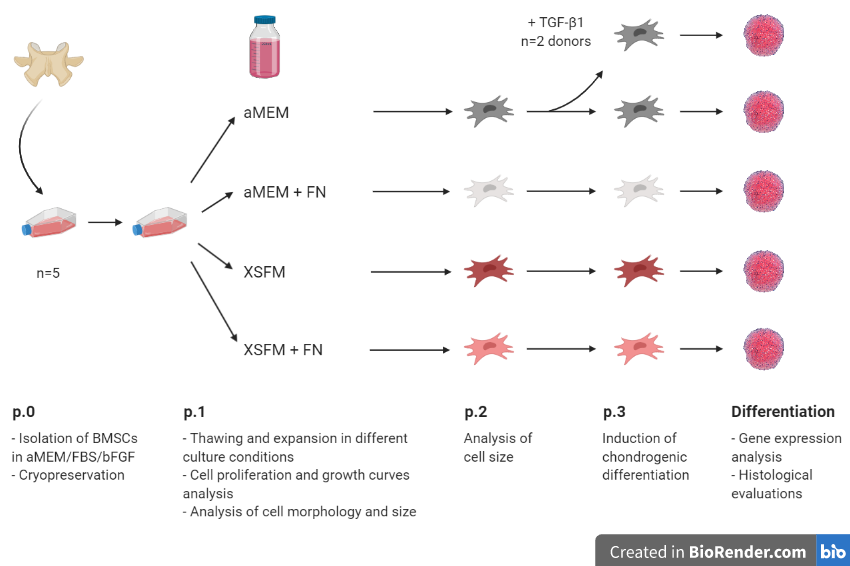
**

Supplement: Supplementary file 1 — Supplementary Information 1. [file 41598_2021_92270_MOESM1_ESM.docx]
