# Supplementary material for: Effect of expansion media and fibronectin coating on growth and chondrogenic differentiation of human bone marrow-derived mesenchymal stromal cells
Source: Sci Rep. 2021 Jun 22;11:13089. doi: 10.1038/s41598-021-92270-4 (PMC8219706; doi:10.1038/s41598-021-92270-4)
Supplement: Supplementary file 2 — Supplementary Information 2. [file 41598_2021_92270_MOESM2_ESM.docx]

**Supplementary Table 1**: List of primers and probes used for qPCR analysis. Custom sequences were synthesized from Microsynth AG (Balgach, Switzerland), while TaqMan assays were purchased from Thermo Fisher. Custom probes are labelled with FAM-TAMRA, while TaqMan assays are labelled with FAM-NFQ-MGB.

| **Target gene** | **Assay ID/sequence** |
| --- | --- |
| **ACAN** | Hs01050178_m1 |
| **COL1A1** | Forward: 5'-CCC TGG AAA GAA TGG AGA TGA T-3' |
|  | Probe: 5'-CGG GCA ATC CTC GAG CAC CCT -3' |
|  | Reverse: 5'-ACT GAA ACC TCT GTG TCC CTT CA-3' |
| **COL2A1** | Forward: 5'-GGC AAT AGC AGG TTC ACG TAC A-3' |
|  | Probe: 5'-CCT GAA GGA TGG CTG CAC GAA ACA TAC-3' |
|  | Reverse: 5'-GAT AAC AGT CTT GCC CCA CTT ACC-3' |
| **COL10A1** | Forward: 5'-ACG CTG AAC GAT ACC AAA TG-3' |
|  | Probe: 5'-ACT ACC CAA CAC CAA GAC ACA GTT CTT CAT TCC-3' |
|  | Reverse: 5'-TGC TAT ACC TTT ACT CTT TAT GGT GTA-3' |
| **IBSP** | Hs00173720_m1 |
| **MMP13** | Forward: 5'-CGG CCA CTC CTT AGG TCT TG-3' |
|  | Probe: 5'-CTC CAA GGA CCC TGG AGC ACT CAT GT-3' |
|  | Reverse: 5'-TTT TGC CGG TGT AGG TGT AGA TAG-3' |
| **PRG4** | Hs00981633_m1 |
| **RPLP0** | Forward: 5'-TGG GCA AGA ACA CCA TGA TG-3' |
|  | Probe: 5'-AGG GCA CCT GGA AAA CAA CCC AGC-3' |
|  | Reverse: 5'-CGG ATA TGA GGC AGC AGT TTC-3' |
| **RUNX2** | Forward: 5'-AGC AAG GTT CAA CGA TCT GAG AT-3' |
|  | Probe: 5'-TGA AAC TCT TGC CTC GTC CAC TCC G-3' |
|  | Reverse: 5'-TTT GTG AAG ACG GTT ATG GTC AA-3' |
| **SOX9** | Hs00165814_m1 |
